# Data from thermal testing of the Open Source Cryostage

**DOI:** 10.1016/j.dib.2016.06.056

**Published:** 2016-07-06

**Authors:** Johannes Lørup Buch, Hans Ramløv

**Affiliations:** a Department of Biology - University of Southern Denmark, Campusvej 55, 5230 Odense M, Denmark; b Department of Science and Environment - Roskilde University, Universitetsvej 1, 4000 Roskilde, Denmark

**Keywords:** Antifreeze proteins, Cryostage

## Abstract

The data presented here is related to the research article “An open source cryostage and software analysis method for detection of antifreeze activity” (Buch and Ramløv, 2016) [1]. The design of the Open Source Cryostage (OSC) is tested in terms of thermal limits, thermal efficiency and electrical efficiency. This article furthermore includes an overview of the electrical circuitry and a flowchart of the software program controlling the temperature of the OSC. The thermal efficiency data is presented here as degrees per volt and maximum cooling capacity.

**Specifications Table**

**Table:** 

Subject area	*Biology*
More specific subject area	*Antifreeze protein research*
Type of data	*Figures*
How data was acquired	*Data was obtained on the OSC, which was developed as a cost efficient alternative to commercial temperature control units and cryostages.*
Data format	*Raw, analysed*
Experimental factors	*Several supply voltages were tested to ensure maximum efficiency.*
Experimental features	*The OSC was tested at increasing supply voltages. The maximum temperature differences over the Peltier Unit were recorded and plotted as absolute numbers, and as a function of voltage.*
Data source location	*n/a*
Data accessibility	*Data is within this article*

**Value of the data**

• The data may serve as a benchmark for further development of the design, or derivatives hereof.
• The data can easily be compared to competing designs, commercial or not.
• The information in this article may inspire others to develop their own designs, something the author highly recommends.

## 1 Data

**Fig. 1:**
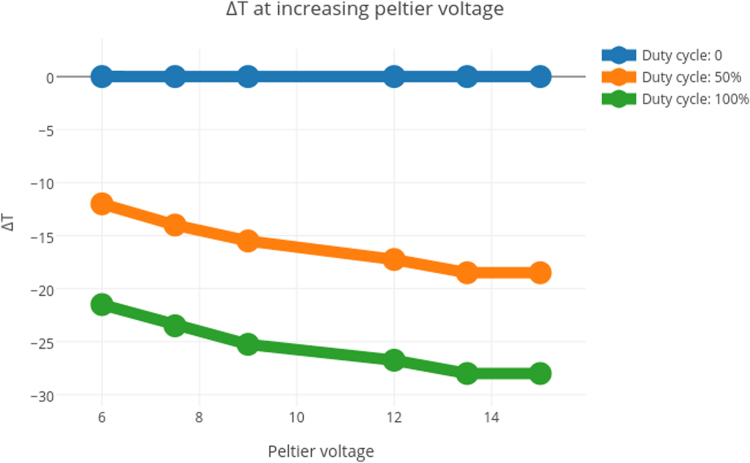
Cooling efficiency of OSC at increasing voltages.

**Fig. 2:**
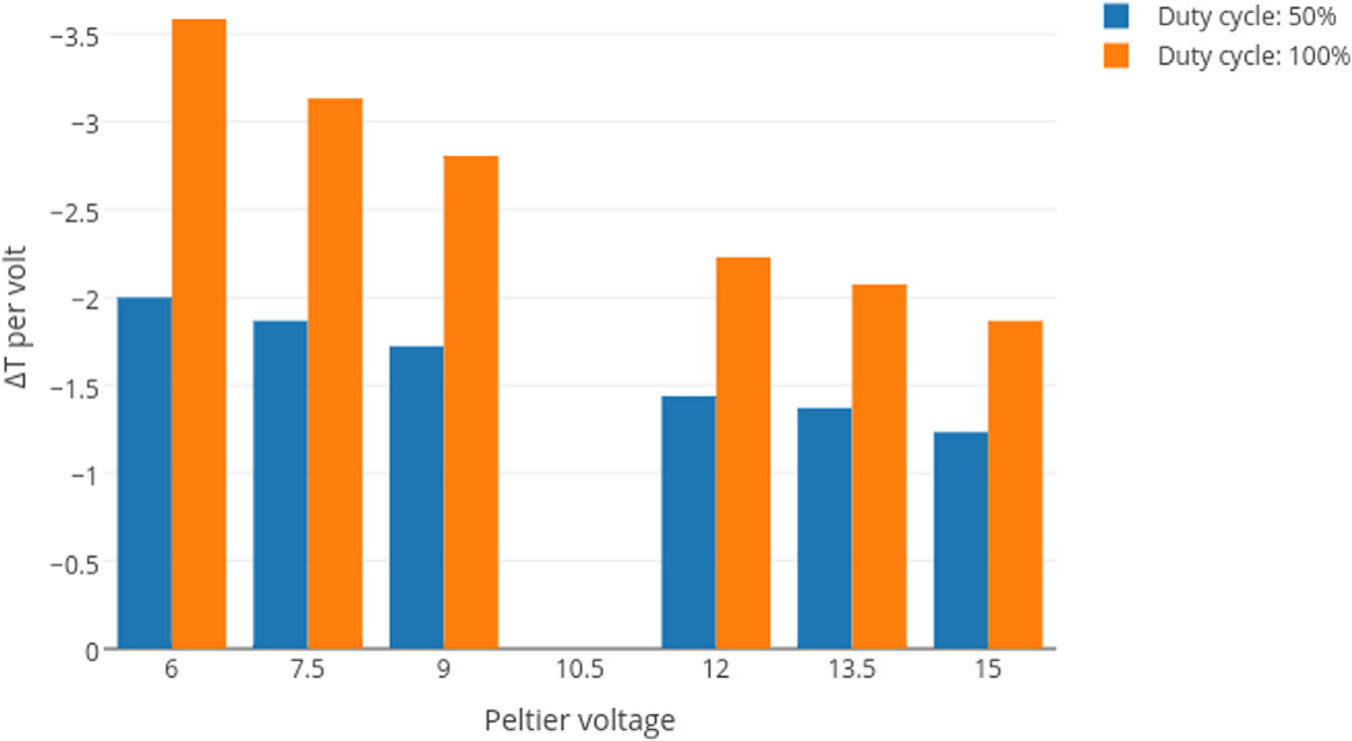
Cooling potential of cryostage at increasing voltages.

**Fig. 3:**
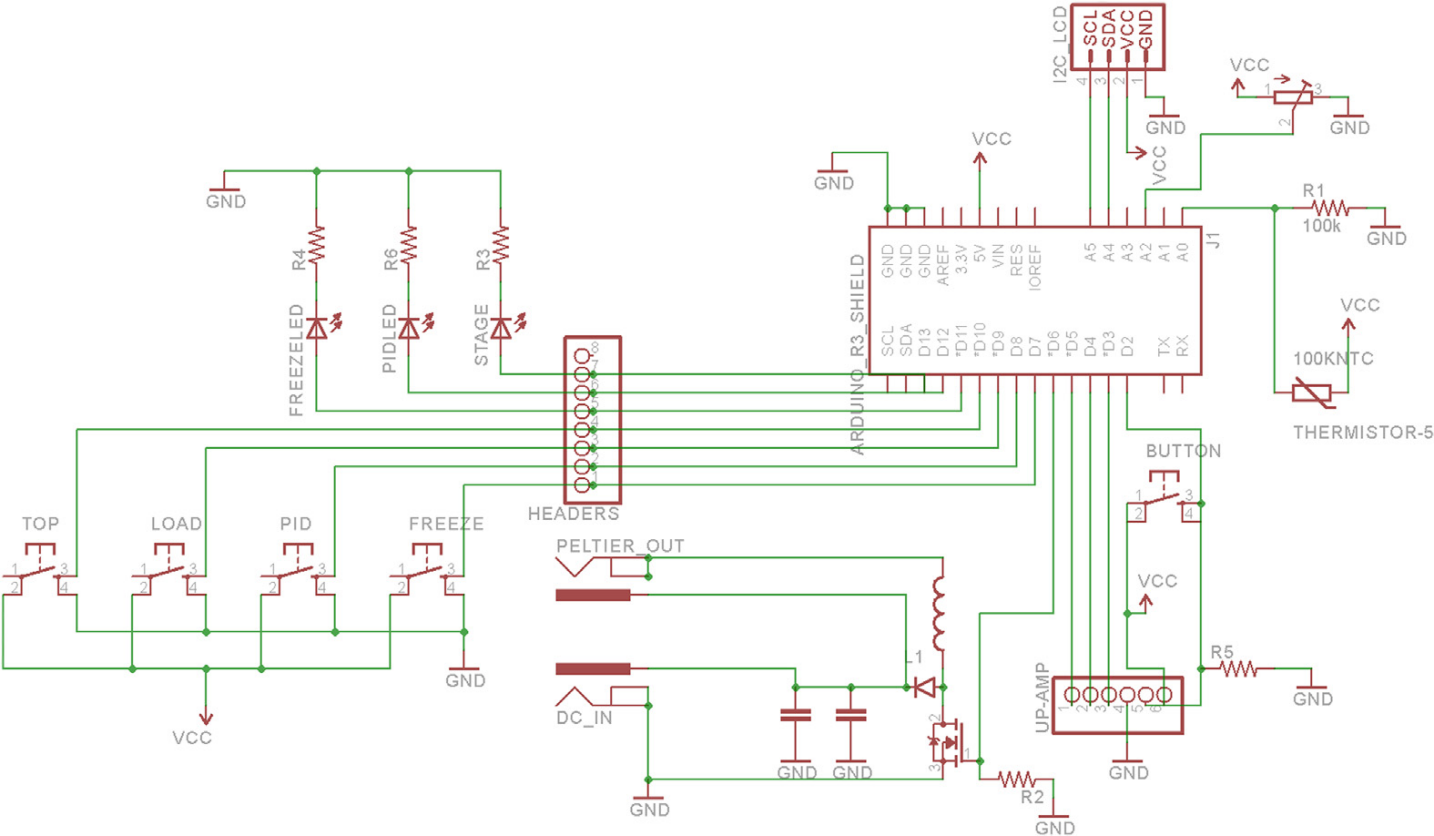
Schematic overview of the Arduino controlled cryostage.

**Fig. 4:**
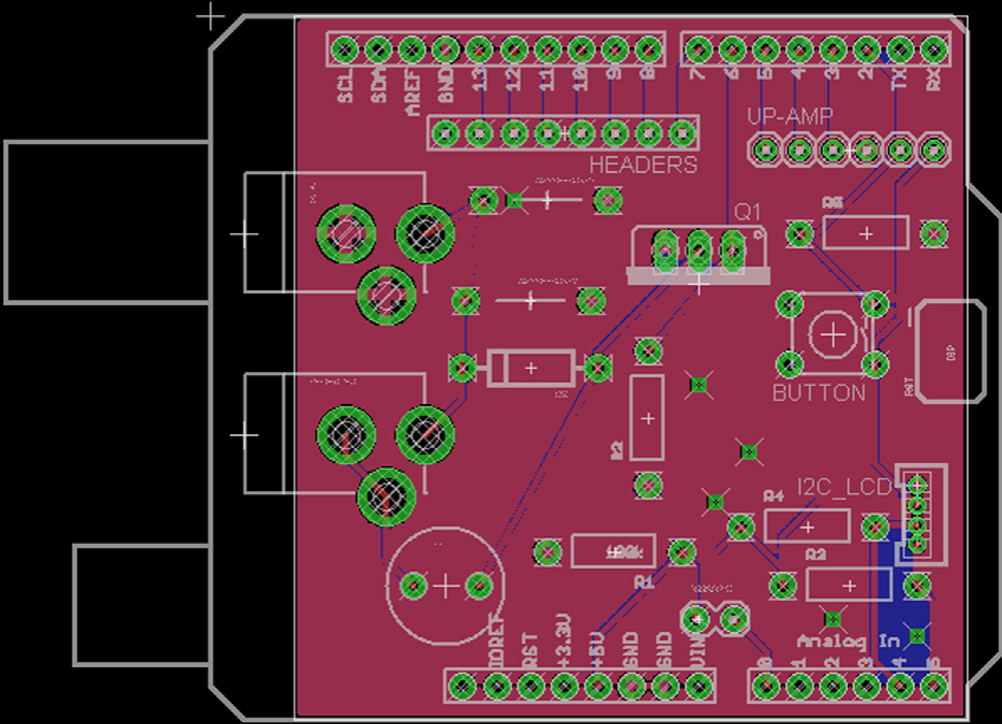
PCB designed for the OSC.

**Fig. 5:**
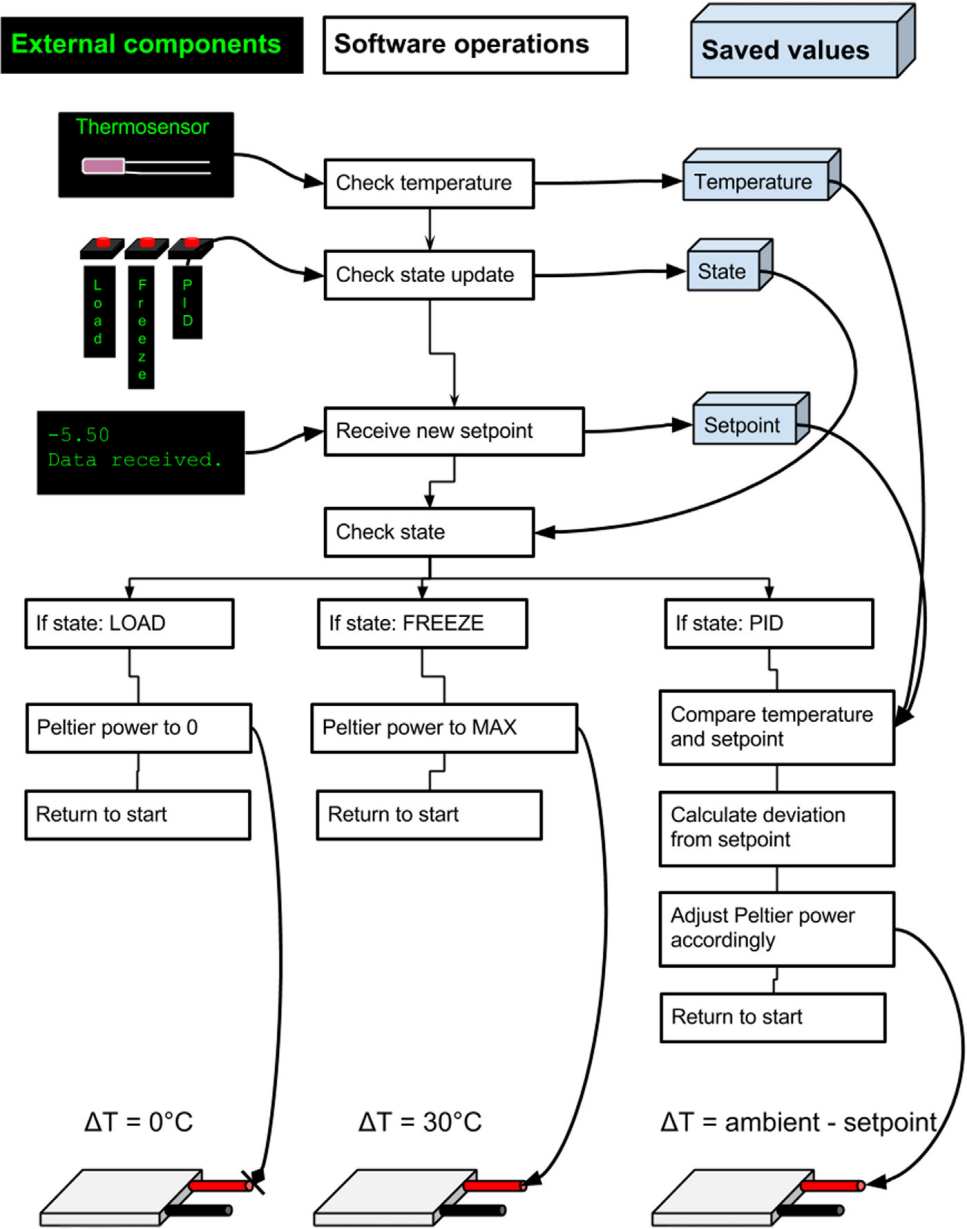
Flowchart showing the temperature control scheme in the OSC. The code is continuously executed from top to bottom.

Fig. 1, Fig. 2 contain the thermal efficiency data. Fig. 3, Fig. 4, Fig. 5 contain information related to the experimental design.

## 2 Experimental design, materials and methods

The OSC׳s cooling capabilities were tested under room temperature conditions, with active air-cooling and a large heatsink. The Peltier unit used in the setup was rated for a max Δ*T* of 60 °C. The power supply was capable of delivering 5 A at varying voltages, all of which were tested. The Peltier module was controlled through PWM, so the duty cycles refer to power draw, relative to maximum.

Several iterations of the OSC were tested before arriving at the design used in this article and the main research article [1]. The electrical design can be seen in Fig. 3.

A custom PCB with the form factor of an Arduino shield was produced from the design seen in Fig. 4.

The OSC has three main settings: LOAD, PID and FREEZE. LOAD will set the temperature of the stage to just below ambient, allowing loading without excessive condensation. FREEZE puts the Peltier unit at maximum power, which will freeze the sample within seconds. PID is the setting which is used during experimentation. A small thermistor provides the Arduino with constant temperature readings, and the Arduino in turn calculates Peltier power level from the temperature deviation from a given setpoint temperature. The setpoint temperature is related to the osmolality of the sample, as described in main article [1]. See Fig. 5 for flow chart.
