# Supplementary material for: Data from thermal testing of the Open Source Cryostage
Source: Data Brief. 2016 Jul 6;8:885–90. doi: 10.1016/j.dib.2016.06.056 (PMC4961764; doi:10.1016/j.dib.2016.06.056)
Supplement: Supplementary file 1 — Supplementary material [file mmc1.doc]

Conflicts of interest:

None
